# Do Maternal Quality of Life and Breastfeeding Difficulties Influence the Continuation of Exclusive Breastfeeding?

**DOI:** 10.1155/2014/156049

**Published:** 2014-04-28

**Authors:** Forough Mortazavi, Seyed Abbas Mousavi, Reza Chaman, Ahmad Khosravi

**Affiliations:** ^1^Department of Midwifery, Faculty of Nursing and Midwifery, Sabzevar University of Medical Sciences, Sabzevar 9613873136, Iran; ^2^Research Center of Psychiatry, Golestan University of Medical Sciences, Golestan 49189 36316, Iran; ^3^Department of Community Medicine, School of Medicine, Yasuj University of Medical Sciences, Yasuj 7591741417, Iran; ^4^Center for Health Related Social and Behavioral Sciences Research, Shahroud University of Medical Sciences, Shahroud 3613773955, Iran

## Abstract

*Objectives.* This study was conducted to determine whether maternal quality of life (QOL) and breastfeeding difficulties influence the continuation of exclusive breastfeeding (EBF). *Methods.* In a survey, 358 consecutive pregnant women filled out a quality of life questionnaire in the third trimester of pregnancy and the breastfeeding experience scale at 4 weeks postpartum. We assessed breastfeeding practices every month up to 6 months postpartum. *Results.* Only 11.8% of women continued EBF at six months. Mothers who continued EBF at 2 and 4 months postpartum had better QOL in late pregnancy than mothers who discontinued it (*P* < 0.05). There were no significant differences between the two groups in QOL scores at 6 months postpartum. Mothers who continued EBF at 2 months postpartum experienced less breastfeeding difficultties during one month postpartum than mothers who discontinued it (*P* < 0.05). *Conclusion.* In attempts to promote EBF, mothers with poor QOL or breastfeeding difficulties in early postpartum should be identified and helped.

## 1. Introduction

There is extensive evidence for short-term and long-term health benefits of breastfeeding for mothers, such as reduced risk of breast and ovarian cancers, and for babies, such as decreased gastroenteritis, respiratory infection, early childhood caries, and diabetes mellitus [1, 2]. The benefits of breastfeeding are maximized when the baby is fed with breast milk exclusively [3]. The World Health Organization recommends that all infants should be fed exclusively on breast milk from birth to six months of age [4].

For many women, breastfeeding is a satisfying and enjoyable experience and they breastfeed successfully despite experiencing breastfeeding difficulties. However, for some women coping with common breastfeeding problems and the recurrent demands of the baby in early postpartum is a physically and emotionally exhausting task [5]. Furthermore, they may have feelings of guilt for not being able to satisfy the baby's needs and harbor doubts about the continuation of breastfeeding, or may even discontinue it [6]. Also, during pregnancy and the first weeks of postpartum, the physical and psychological health of the mother goes through considerable changes. Most mothers experience symptoms, which may influence the mother's physical and emotional health [7]. A previous study has found that maternal depressive symptoms in early postpartum were related to breastfeeding experience [8]. Results of another study revealed that the mother's breastfeeding experience is affected by multiple factors, such as psychological and physical health, sociodemographic characteristics, quality of marital relationship, and living condition [9]. Quality of life (QOL) is a broad ranging concept that includes all the mentioned aspects [10]. Despite the potential role of maternal quality of life (QOL) in the breastfeeding experience, there is limited scientific evidence about the relationship between QOL and breastfeeding continuation. A Brazilian study reported that QOL was correlated with breastfeeding self-efficacy in postpartum [11]. In a cross-sectional study in Taiwan, QOL was related to breastfeeding continuation [12].

The Iranian government has successfully promoted breastfeeding through policy change. In 2007, the rate of breastfeeding in the country at first 2 years of age was reported at 57% [13]. Although Iran has a high rate of breastfeeding initiation and continuation, its rate of exclusive breastfeeding (EBF) is decreasing. The rates of EBF at sixth months of age were 44% in 2000 and 23% in 2010 [14]. Studies conducted in Iran have reported that difficulties such as sore nipples, latching-on problems, and the mother's perception of having insufficient milk or her perception of insufficient infant weight gain were the reasons for the discontinuation of breastfeeding [15, 16].

The present study aims to investigate the relationships between QOL and breastfeeding difficulties and the continuation of EBF in a sample of mothers from northeast of Iran. The use of WHOQOL-BREF,which covers four domains of QOL (somatic, psychological, social, and environmental) allowed us to overcome a shortcoming of previous studies, which have generally focused on somatic and psychological domains. In addition, previous studies have investigated breastfeeding difficulties using questionnaires, which allow only yes or no responses or assess difficulties separately. In the present study, we assessed breastfeeding difficulties using BES, which was developed to measure common breastfeeding difficulties in the form of a continuous variable in the postpartum and to assess the total breastfeeding difficulties. Moreover, the BES includes multiple factors related to infant and mother, which allowed us to measure difficulties more multidimensionally. To the authors' best knowledge, this is the first study to explore the relationship between maternal QOL and breastfeeding difficulties and the continuation of EBF using these questionnaires.

## 2. Methods

### 2.1. Study Design and Participants

This survey was started in May 2011 in Shahroud, a city in northeast Iran. We selected 370 consecutive women, who attended 10 urban health clinics, affiliated to Shahroud University of Medical Sciences, for prenatal care and met the inclusion criteria over a period of six months. We chose health clinics as our research environment because 94% of pregnant women in Iran have at least 4 visits to health clinics during pregnancy [16]. Three hundred and fifty eight of the women agreed to participate in the study and gave informed consent, of which 347 were followed up until 6 months postpartum. Midwives at the health clinics were responsible for distributing and collecting questionnaires and assessing infant feeding methods.The inclusion criteria were gestational age of at least 28 weeks and no serious medical condition that prevented breastfeeding. The exclusion criteria were fetal death, infant death, infant major abnormalities and serious medical condition that prevented breastfeeding, and twin pregnancy. The participants completed the WHOQOL-BREF in the third trimester of pregnancy. Women completed the BES at 4 weeks postpartum. Shahroud University of Medical Center Institutional Review Board approved this study. All procedures followed were in accordance with the ethical standards of the Ethics Committee of the Shahroud University of Medical Sciences (approval number 900.02).

### 2.2. Instruments

#### 2.2.1. Sociodemographic and Obstetrical Questionnaire

Women completed a questionnaire consisting of sociodemographic information (age, education, income, and occupation) at the first visit. They completed another questionnaire consisting of obstetrical information (parity, pregnancy wantedness, mode of delivery, BMI, pregnancy weight gain, weight at last prenatal visit, and previous breastfeeding experience) at the 2 weeks postpartum.

### 2.3. Infant Feeding Checklist

Midwives assessed the infant feeding method at 2 and 4 weeks postpartum and then every month up to 6 months by asking mother to list all fluids and foods consumed by the infant (including breast milk, formula, other fluids, semisolids, and solids) during the month previous to the face-to-face interview using a check list. EBF were defined according to WHO definitions [4]. The 2nd, 4th, 8th, 16th, and 24th weeks of postpartum coincide with women's scheduled clinic visits. Midwives made phone calls at 12th and 20th weeks postpartum to assess the infant feeding method.

### 2.4. WHOQOL-BREF

The WHOQOL-BREF was developed by the World Health Organization [10]. It contains 24 questions divided into 4 domains: physical (7 questions), psychological (6 questions), social relationships (3 questions), and environment (8 questions). There are also two more questions: question 1 asks about an individual's overall perception of quality of life and question 2 asks about an individual's overall perception of his or her health. The items are rated on a 5-point Likert scale. We transformed the raw domain scores to a 0–100 scale. In addition, we calculated the mean score of all 26 questions and then transformed the global score of QOL to a 0–100 scale. The validity and reliability of the Iranian version of WHOQOL-BREF have been supported in a previous study [17]. The validity of the questionnaire among women in the postpartum period has also been supported by previous studies [18, 19].

### 2.5. Breastfeeding Experience Scale

The breastfeeding experience scale (BES) is a questionnaire consisting of 30-items, which measure breastfeeding methods, experiences, and outcomes. We only used the first 18 questions of this 30-item scale which rate the severity of common breastfeeding difficulties in the early postpartum period using a 5-point Likert scale (1 = not at all and 5 = unbearable). The scale assesses if these problems ever occurred. Items are as follows: sore nipples, cracked nipples, leaking breasts, breast engorgement, breast infection, baby having sucking difficulty, baby having difficulty in latching on, baby reluctant to nurse due to sleepiness, baby reluctant to nurse due to fussiness, baby nursing too frequently, feeling very tired, feeling tense and overwhelmed, worry about not having enough milk, worry that baby was not getting enough milk, difficulty positioning baby, worry about baby's weight gain, feeling embarrassed when nursing, and difficulty combining work and breastfeeding. Responses are then summed up to obtain a total breastfeeding difficulties score (range of 18–90), with a higher score representing increased problem severity. Content validity and internal consistency of this scale (alpha coefficient 0.76) have been reported and supported in a previous study [20]. In our study, the alpha coefficient was 0.82 at 4 weeks postpartum. We translated the instrument into Persian and a PhD in English language back-translated the instrument into English. We compared the back-translated version with the original instrument and found no discrepancy in items' meaning. Then a panel of experts in obstetrics and pediatrics assessed the instrument. We changed no item.

### 2.6. Breastfeeding Intention

Intention to breastfeed was assessed by a question using a 5-point numerical rating scale (1 = definitely breastfeed and 6 = definitely not breastfeed)

### 2.7. Statistical Analysis

Statistical analyses were performed using Spss 18 (SPSS Inc., Chicago, IL, USA). The results are reported as means and standard deviations for continuous variables and as frequencies and percentages for categorical variables. Differences between exclusive and nonexclusive breastfeeding groups were assessed using* t*-tests for continuous variables and chi-square test for categorical variables. The significance level of tests was set at 0.05.

## 3. Results

### 3.1. Women's Characteristics

A total of 358 women participate in the study of which 347 were followed up until 6 months postpartum. Mean age of women was 26.17 ± 4.42. Median monthly household income was 4 million RlS. The educational levels of women were primary school 11%, secondary school 61%, and university 28%. None of them smoked or had a history of smoking or alcohol dependence. All were married. About 28% of women described their previous breastfeeding experience as excellent or good and 4.5% as moderate.  Table 1 shows women's characteristics based on breastfeeding method at 2 and 4 months postpartum. The relative risk of exclusive breastfeeding discontinuation at 2 months postpartum was 1.4 times higher for women who had no breastfeeding experience than women who had previous breastfeeding experience.  Table 2 shows the frequency of different breastfeeding methods at 2, 4, and 6 months postpartum. The value of Alpha Cronbach coefficient for the whole WHOQOL-BREF questionnaire was 0.92.

### 3.2. QOL

The two first independent questions of WHOQOL-BREF assess woman's overall perception of quality of life and her health, respectively. 27% of women evaluated their overall quality of life as “very good,” 54% as “good,” 18% as “not good, not bad,” 8% as “bad,” and 0.3% as “very bad.” Also, 29% of women evaluated their overall health as “very good,” 52% as “good,” 16% as “not good not bad,” 1.7% as “bad,” and 1.7% as “very bad.”

### 3.3. Breastfeeding Difficulties

The mean total score of the breastfeeding difficulties scale (i.e., the score for the first 18 items of BES) was 31.4 ± 8.5 with range of 18–74. The value of Alpha Cronbach coefficient for the breastfeeding difficulties scale was 0.83. Common difficulties experienced by women were baby's frequent demand for breastfeeding (81.6%), leaking breasts (78.2%), difficulty combining work and breastfeeding (69%), feeling exhausted (59%), and worry about having enough milk (52%). The mean scores of the breastfeeding difficulties questionnaire for primigravidas and multigravidas were 32.32 ± 9.7 and 30.1 ± 6.4, respectively (*P* = 0.012).

### 3.4. QOL and Breastfeeding Difficulties and the Continuation of Exclusive Breastfeeding

Table 3 shows the scores of QOL and breastfeeding difficulties for women with exclusive and nonexclusive breastfeeding at 2 and 4 months postpartum. There were no significant differences between the two groups in QOL and breastfeeding difficulties scores at 6 months postpartum.

## 4. Discussion

In this study, we examined the relationship between QOL and breastfeeding difficulties during 4 weeks postpartum and the continuation of EBF in women visiting urban health clinics in Shahroud, Iran. Our results indicate that mothers who continued EBF at 2 and 4 months postpartum had better QOL in late pregnancy than mothers who discontinued EBF. These findings are comparable with a Brazilian research, which found a correlation between breastfeeding self-efficacy and maternal QOL [11]. It is possible that maternal QOL improved after childbirth gradually due to improvement in mother's physical and emotional health. Two months postpartum is the period in which physiological changes resulting from pregnancy recede and the body returns to its normal condition. Also, during the same period the postpartum mood fluctuations disappear. However, mothers with low QOL in late pregnancy may undergo the transitional period more slowly than mothers with higher QOL. This difference may account for the failure to continue EBF in mothers with a low QOL. On the other hand, the rate of complementary breastfeeding at 4 and 6 months postpartum were 3.2% and 45.8%, respectively. This means that more than 40% of mothers discontinue EBF during 5th and 6th months postpartum. This may be due to traditional beliefs about infant feeding with its emphasis on introducing semisolid foods after 4 months postpartum rather than a low QOL. This may account for the similarity of 2 groups in the QOL scores at 6 months postpartum. In other words, since the introduction of solid and semisolid foods was a common practice during the fifth and sixth months postpartum in this study, antepartum QOL could not affect the continuation of EBF at 6 months. In addition, mothers who continued EBF at 2 months postpartum experienced lower breastfeeding difficulties during first month postpartum than mothers who discontinued it. There were no significant differences between the 2 groups in breastfeeding difficulties scores at 4 and 6 months postpartum. Breastfeeding difficulties are common and transient during early postpartum [21]. Results of a previous study revealed that breastfeeding difficulty and severity decrease from 3 days to 9 weeks postpartum [22]. Therefore, the breastfeeding difficulty at 4 weeks postpartum may not account for EBF discontinuation at 4 and 6 months postpartum. Previous qualitative research indicated that obstacles or problems, such as perceptions of insufficient milk supply, nipple/breast pain, difficulty combining work and breastfeeding, problems with pumping, and feeling overwhelmed and frustrated, led adolescent mothers to weaning [23]. In our study, baby nursing too frequently, leaking breasts, difficulty combining housekeeping and breastfeeding, feeling very tired or fatigued, and worry about having enough milk were the most frequent major breastfeeding difficulties identified by women. In a study, painful nipples/breasts, low milk supply, and latching difficulties were the three most frequent major breastfeeding problems identified by women [24].

In this study, the rates of EBF were 28.8%, 23%, and 11.8% at 2, 4, and 6 months postpartum, respectively. The sampling site represents its own local characteristics and could not represent national status. In fact, previous studies in Iran have variously reported the rate of EBF at 6 months at 27.7%, 52.6%, and 33.1% [13, 16, 25]. The differences may be due to the different approaches to data collection methods and study design. Our study was longitudinal and we asked mothers to recall what their babies had eaten during last month while the cited cross-sectional studies had used the 24 hours recall method to collect the data related to infant feeding. Therefore, we could identify four breastfeeding groups (exclusive, predominant, partial, and complementary), while other studies mentioned three groups (exclusive, partial, and complementary).

In relation to women's QOL, the majority of women in this study (81%) described their QOL as “good” or “very good” according to the first independent question of WHOQOL-BREF and 81% were “satisfied” with their health status according to the second independent question of the WHOQOL-BREF. Results of a Brazilian study on maternal postpartum QOL indicated that 70.8% of mothers rated their overall QOL as “very good” or “good” [11].

We could find a negative relationship between educational attainment and EBF. In a previous study conducted in Peru, maternal education was negatively associated with EBF at 3 months [26], while in a study on Icelandic women, educational attainment was an important factor in EBF continuation [27].

As expected, multiparous women had less breastfeeding difficulties. This is due to their previous experience in breastfeeding. Eighty percent of multiparous women described their breastfeeding experience as good or excellent.

This study increased our knowledge about maternal QOL and breastfeeding difficulties in Shahroud and contributed to our understanding of the relationship between QOL and breastfeeding difficulties and the continuation of exclusive breastfeeding. We recommend that further studies be designed to assess the relationship between QOL, breastfeeding difficulties and breastfeeding discontinuation. Since 62.8% of our sample adopted predominant breastfeeding up to 2 months postpartum, and breastfeeding intention was not different in EBF and non-EBF groups, we recommend that qualitative research be designed to investigate the other reasons of discontinuation of EBF in early postpartum.

In this study, the QOL questionnaire was conducted only in the third trimester of pregnancy, so it is possible that childbirth and breastfeeding improved mother's perception of wellbeing and the QOL. We recommend that further studies be designed to assess the trajectory of changes in the maternal QOL during the first six months postpartum and its influence on breastfeeding practices and duration.

## 5. Limitations

The sample in this study was representative of low risk Iranian women visiting public health clinics to receive prenatal care in Shahroud. Only 32 women did not choose to participate in our study due to lack of time to fill out the questionnaire. Limitation of our study includes potential for recall bias. We acknowledge that the method used to assess breastfeeding outcomes may lead to misclassifications. Since the 24-hour recall method overestimates EBF rates, we chose a longer recall time (the month recall). Also, we asked mothers to recall breastfeeding difficulties during 4 weeks postpartum, so recall bias is possible.

## 6. Conclusion

Breastfeeding difficulties and QOL could affect the continuation of exclusive breastfeeding. To promote EBF, mothers with low QOL or breastfeeding difficulties should be supported during the early postpartum.

## Figures and Tables

**Table 1:** Women's characteristics based on breastfeeding practices.

Variable	2 months postpartum			4 months postpartum		
	EBF^†^	Non-EBF		EBF	Non-EBF	
	*N* (%) or M ± SD		*P*	*N* (%) or M ± SD		*P*
Age	26.3 ± 4.6	26.2 ± 4.3	0.870	26.7 ± 4.8	26.1 ± 4.3	0.297
Education	10.7 ± 4.2	11.7 ± 3.5	0.031*	10.6 ± 4.2	11.6 ± 3.6	0.063
Income	431 ± 165	423 ± 146	0.688	430 ± 170	424 ± 146	0.785
BMI	23.4 ± 3.7	24.4 ± 4.6	0.129	23.5 ± 3.6	24.3 ± 4.5	0.264
Occupation						
Yes	12 (37.5)	20 (62.5)	0.291	9 (28.1)	23 (71.9)	0.502
No	90 (28.6)	225 (71.4)		72 (22.9)	243 (77.1)	
Mode of delivery						
Cesarean	54 (30.3)	124 (69.7)	0.719	47 (26.4)	131 (73.6)	0.176
Vaginal delivery	48 (28.6)	120 (71.4)		34 (20.2)	134 (9.8)	
Parity						
Primiparity	52 (25.7)	150 (74.3)	0.078	40 (19.8)	162 (80.2)	0.066
Multiparity	49 (34.5)	96 (65.5)		41 (28.3)	104 (71.7)	
Desirability of pregnancy						
Yes	88 (28.9)	217 (71.1)	0.706	69 (22.6)	236 (77.4)	0.549
No	13 (31.7)	28 (68.3)		11 (26.8)	30 (73.2)	
Health						
Healthy	98 (30.1)	228 (69.9)	0.602	79 (24.2)	247 (75.8)	0.263
Patient	4 (22.2)	14 (77.8)		2 (11.1)	16 (88.9)	
Postpartum rehospitalization						
Yes	7 (35)	13 (65)	0.589	6 (30)	14 (70)	0.322
No	95 (29.3)	229 (70.7)		75 (23.1)	249 (76.9)	
Previous breastfeeding experience						
Yes	47 (36.7)	81 (63.3)	0.022*	38 (29.7)	90 (70.3)	0.033*
No	55 (25.1)	164 (74.9)		43 (19.6)	176 (80.4)	

*P < 0.05; *t*-test; chi-square test; Fisher's exact test; ^†^exclusive breastfeeding.

**Table 2:** Frequency of different breastfeeding practices.

Breastfeeding methods	2 months postpartum	4 months postpartum	6 months postpartum
Exclusive breastfeeding^†^	100 (28.8%)	80 (23.0%)	41 (11.8%)
Predominant breastfeeding^‡^	211 (60.8%)	218 (62.8%)	97 (28%)
Partial feeding^*δ*^	34 (9.8%)	33 (9.5%)	40 (11.5%)
Complementary feeding^§^	—	11 (3.2%)	159 (45.8%)
Formula feeding	2 (0.6%)	5 (1.4%)	10 (2.9%)

^†^Breast milk; ^‡^breast milk and water based fluid; ^*δ*^breast milk and nonhuman milk or formula; ^§^breast milk and any solid or semisolid foods or liquid.

**Table 3:** Means and standard deviations of the quality of life score, breastfeeding difficulties score, and intention to breastfeeding based on breastfeeding practices.

2 months postpartum	Exclusive breastfeeding (*N* = 100)	Nonexclusive breastfeeding (*N* = 247)	*P* value
	M ± Sd	M ± Sd	
Domains of quality of life			
Physical	66.7 ± 17.3	61 ± 16.9	0.005**
Mental	66.3 ± 18.5	62.3 ± 15.7	0.040*
Social	67.7 ± 20.7	65.5 ± 20.5	0.366
Environmental	69.7 ± 14.9	68.4 ± 14.6	0.450
Global	68.5 ± 14.8	65.2 ± 13.1	0.042*
Breastfeeding difficulties score	29.71 ± 7.3	8.9 ± 32.12	0.017*
Intention to breastfeeding	5.8 ± 0.6	5.7 ± 0.7	0.714

4 months postpartum	Exclusive breastfeeding (*N* = 80)	Nonexclusive breastfeeding (*N* = 267)	

Domains of quality of life			
Physical	67.2 ± 17.4	61.3 ± 17.5	0.001**
Mental	66.8 ± 18.9	62.5 ± 15.7	0.013*
Social	67.5 ± 21.9	66.1 ± 20.1	0.604
Environmental	69.0 ± 14.9	68.8 ± 14.6	0.720
Global	68.4 ± 15.1	65.5 ± 11.2	0.025*
Breastfeeding difficulties score	29.9 ± 7.1	31.9 ± 8.9	0.063
Intention to breastfeeding	5.8 ± 0.6	5.7 ± 0.7	0.323

*P < 0.05; **P < 0.01; *t*-test.
